# Understanding academic resilience over time through psychological resources and social support in the Yangtze River Delta region of China

**DOI:** 10.3389/fpsyg.2026.1793326

**Published:** 2026-04-22

**Authors:** Xingtong Yu, Mansor Abu Talib, Dan Wei

**Affiliations:** 1 Faculty of Social Sciences and Liberal Arts, UCSI University, Kuala Lumpur, Malaysia; 2 Wuhan Donghu University, Wuhan, Hubei, China; 3 Wellbeing Research Centre, UCSI University, Kuala Lumpur, Malaysia

**Keywords:** academic resilience, motivation, self-efficacy, self-esteem, social support

## Abstract

**Introduction:**

Academic resilience is an important psychological resource that enables university students to cope with academic challenges and persist in demanding learning environments. Drawing on Social Cognitive Theory, this study examined the combined roles of social support, self-efficacy, self-esteem, and motivation in predicting academic resilience among university students in the Yangtze River Delta region of China.

**Methods:**

A three-wave time-lagged design was employed, with data collected over three consecutive academic semesters from a final analytical sample of 2,609 undergraduate and postgraduate students from the provinces of Zhejiang, Shanghai, and Jiangsu. A three-wave time-lagged structural equation modelling approach was used to examine whether earlier social support and psychological resources were prospectively associated with later academic resilience, while controlling for age, gender, and education level.

**Results and discussion:**

The results indicated that self-efficacy was the strongest predictor of academic resilience, followed by motivation and social support, all of which showed significant positive effects. Self-esteem also showed a positive association overall, although its effect varied across regions. Among the control variables, age and education level were significantly associated with academic resilience, whereas gender was not significant in the general model. Regional analyses further revealed meaningful variations in the strength and significance of the predictors across provinces. These findings highlight the importance of integrating psychological and environmental resources within a unified theoretical framework and underscore the role of contextual factors in shaping academic resilience. The study contributes to the academic resilience literature by providing evidence of prospective, time-lagged associations among key psychological and contextual factors and by offering practical implications for designing targeted interventions to strengthen students’ adaptive capacity in higher education.

## 1 Introduction

Academic resilience refers to students’ capacity to sustain academic engagement, adapt effectively to challenges, and recover from setbacks in demanding educational contexts. Within higher education, academic resilience has become an important concern as universities increasingly confront rising academic pressure, competitive learning environments, and the growing prevalence of stress-related psychological difficulties among students. University students are expected to manage complex academic tasks, regulate learning autonomously, and navigate uncertainty associated with academic progression and future employment. These demands frequently coincide with developmental transitions and social adjustments, increasing vulnerability to academic disengagement and psychological strain (Brewer et al., 2019). In this context, academic resilience may be understood as an adaptive capacity shaped by both personal and environmental resources, making temporally ordered investigation important.

Evidence suggests that insufficient academic resilience is associated with adverse outcomes, including emotional exhaustion, reduced motivation, diminished academic performance, and heightened risk of withdrawal or dropout (Qi, 2025; Shengyao et al., 2024a). Students who experience repeated academic setbacks without adequate coping resources may develop maladaptive responses such as avoidance, helplessness, or disengagement. Conversely, resilient students tend to interpret academic difficulty as manageable, sustain effort during periods of challenge, and re-engage following failure. Although academic resilience is often discussed as developing across educational experiences, understanding it empirically requires designs that can examine whether earlier psychological and contextual resources are associated with later adaptive functioning.

Prior research conceptualises academic resilience as the product of both personal and environmental factors. Personal resources include psychological characteristics that influence how students interpret academic demands and regulate behavior in response to stress. Among these, self-efficacy, motivation, and self-esteem have been consistently associated with adaptive academic functioning. Self-efficacy reflects students’ beliefs about their capability to succeed in academic tasks and is strongly linked to persistence, effort regulation, and recovery from failure (Bandura, 2001). Motivation captures the direction, intensity, and persistence of academic goal pursuit and is essential for sustaining engagement across prolonged academic challenges. Self-esteem reflects students’ broader sense of self-worth within academic contexts and contributes to emotional stability and confidence when encountering difficulty. Prior longitudinal research suggests that these psychological resources may be associated with subsequent academic adaptation, highlighting the value of temporally ordered investigation (Tan et al., 2024).

Environmental resources, particularly social support, further shape academic resilience by buffering stress and reinforcing adaptive coping. Support from peers, family members, and educational institutions can provide emotional reassurance, practical assistance, and informational guidance, enabling students to persist during academically demanding periods. Social support may also strengthen psychological resources by enhancing students’ confidence, motivation, and sense of belonging (Suud et al., 2024; Zarza-Alzugaray et al., 2020). Because the availability and perceived usefulness of support may vary across stages of university life, a temporally ordered design is helpful for examining whether earlier support is associated with later academic resilience.

Despite extensive empirical attention, the academic resilience literature presents notable limitations. Many studies rely on cross-sectional designs that capture associations at a single time point, limiting insight into temporal ordering among variables. Such designs cannot adequately determine whether psychological and contextual resources precede resilience-related outcomes or are simply correlated with them. Furthermore, prior research often examines resilience predictors in isolation or in partial combinations, yielding fragmented findings that obscure the joint operation of psychological and social factors. Integrated, theory-driven models that examine how multiple resources are prospectively associated with academic resilience remain relatively scarce (Brewer et al., 2019).

Contextual considerations further complicate the study of academic resilience. Much of the existing literature treats student populations as relatively homogeneous, with limited attention to socio-cultural and regional variation. In large and diverse educational systems such as China’s, regional differences in academic culture, institutional resources, and social expectations may substantially influence how students experience academic stress and mobilise coping resources. These contextual influences are especially relevant when examining academic resilience in temporally ordered models, as educational environments may shape the availability and impact of psychological and social resources over time. Yet, research that combines temporal ordering with regional context remains limited.

Demographic factors such as age, gender, and education level have also shown inconsistent associations with academic resilience. Some studies suggest that older students or those at more advanced educational stages may demonstrate higher resilience due to accumulated experience and self-regulatory skills, whereas others report increased vulnerability associated with heightened academic demands (Martin and Marsh, 2006). Gender differences have likewise produced mixed findings, indicating that demographic influences may depend on cultural, institutional, or developmental contexts. A multi-wave design offers a useful approach for examining whether these demographic characteristics are associated with later academic resilience alongside psychological and environmental factors.

SCT provides a useful framework for addressing these gaps. The theory emphasises the interaction of personal factors, environmental influences, and behavioral outcomes, viewing individuals as proactive agents in their own functioning and adaptation (Bandura, 2001). Within this framework, self-efficacy plays a central role in shaping how individuals approach challenges, regulate effort, and persist in the face of adversity. Applied to academic contexts, SCT supports an integrated view in which self-efficacy, self-esteem, motivation, and social support may together help explain later academic resilience. Although the theory recognizes reciprocal influences conceptually, the present study uses SCT to justify a prospective time-lagged framework rather than to test fully reciprocal or feedback processes directly.

The present study is situated in the Yangtze River Delta region of China, one of the country’s most economically developed and educationally competitive areas. The region encompasses Zhejiang, Shanghai, and Jiangsu, which share high academic expectations and strong institutional capacity while differing in levels of urbanization, educational culture, and student experiences. These characteristics make the Yangtze River Delta a compelling context for examining academic resilience under sustained academic pressure. Investigating resilience in this region makes it possible to examine whether psychological and social resources show similar or varying prospective associations with academic resilience across different provincial contexts.

Against this backdrop, the current study uses a three-wave time-lagged perspective to examine whether self-efficacy, motivation, self-esteem, and social support are prospectively associated with academic resilience among university students in the Yangtze River Delta region of China. Drawing on SCT, the study integrates personal and environmental resources within a unified analytical framework and controls for demographic factors. By moving beyond cross-sectional analysis and situating academic resilience within a specific regional context, the study aims to examine temporally ordered prospective associations related to academic resilience. The findings are expected to contribute theoretically by clarifying time-lagged relationships among key predictors and practically by informing interventions that support students’ adaptive capacity in higher education.

## 2 Literature review and hypothesis development

### 2.1 Self-efficacy

Self-efficacy refers to students’ beliefs in their ability to organise and execute the actions required to meet academic demands. Within SCT, self-efficacy is central because it shapes how students interpret challenges, regulate effort, and persist in the face of setbacks (Bandura, 2014). In higher education, students with stronger academic self-efficacy are more likely to approach difficult tasks as manageable, remain engaged under pressure, and recover more effectively from academic difficulties. Recent evidence continues to support this role. Shengyao et al. (2024a) found that self-efficacy was significantly associated with academic resilience and served as the strongest mediator in their model. Similarly, Wang and Zhang (2024) reported that academic self-efficacy was positively related to psychological resilience and learning engagement among college students, while Bagdžiūnienė et al. (2025) found that self-efficacy positively predicted academic resilience among university students and showed a stronger effect than study-related resources. Therefore, self-efficacy is expected to be positively associated with academic resilience.

### 2.2 Self-esteem

Self-esteem refers to individuals’ overall evaluation of their personal worth and value. In the university context, self-esteem influences how students interpret setbacks, regulate emotional responses, and maintain confidence when facing academic pressure. Students with higher self-esteem are generally more likely to cope with difficulties constructively, whereas those with lower self-esteem may be more vulnerable to discouragement and withdrawal. Recent higher-education research supports this role. Ni et al. (2025) found that self-esteem positively predicted college students’ learning adaptation, operating through self-efficacy and reduced learning burnout. In addition, Shang et al. (2025) reported that self-esteem significantly and positively predicted resilience among university students. Taken together, these findings suggest that self-esteem functions as an important affective resource that can support adaptive coping and resilient academic functioning. Accordingly, self-esteem is expected to be positively associated with academic resilience.

### 2.3 Motivation

Motivation refers to the processes that energise, direct, and sustain goal-oriented behavior. In academic settings, motivation influences whether students invest effort, remain engaged, and continue working toward educational goals despite stress or obstacles. From the perspective of academic resilience, motivation is important because resilient students must not only recover from setbacks but also sustain effort and direction when learning becomes demanding. Recent studies support this connection. Shengyao et al. (2024a) showed that academic motivation was significantly associated with academic resilience within a SCT framework. Likewise, Melaku et al. (2025) found significant positive associations among perceived academic support, academic motivation, academic resilience, and academic achievement among first-year university students, with motivation and resilience jointly mediating the effect of support on achievement. These findings suggest that motivation helps transform support and personal resources into sustained academic adaptation and persistence. Therefore, motivation is expected to be positively associated with academic resilience.

### 2.4 Social support

Social support refers to the emotional, informational, and instrumental assistance students receive from important others, including family members, peers, and teachers. In higher education, social support is a key contextual resource because it can reduce isolation, strengthen belonging, and provide encouragement and practical help during periods of academic stress. Recent evidence suggests that support is relevant not only directly, but also through its influence on personal resources. Zhang et al. (2024) found that social support was positively associated with both self-efficacy and resilience among vocational college students within a SCT model. Melaku et al. (2025) likewise reported that perceived academic support was positively associated with academic motivation and academic resilience among first-year university students. In addition, a recent university-student study concluded that social support functioned as both a direct predictor of academic resilience and an indirect predictor through positive affect. These findings suggest that supportive relationships can strengthen both students’ coping capacity and the personal resources needed for persistence under difficulty. Thus, social support is expected to be positively associated with academic resilience.

### 2.5 Social cognitive theory

The present study adopts SCT as its core theoretical framework for explaining academic resilience among university students. SCT proposes that human functioning is shaped by the interaction of personal factors, environmental influences, and behavioral outcomes (Bandura, 1986; Bandura et al., 1977). From this perspective, academic resilience is not viewed as a fixed trait, but as an adaptive outcome that develops through the combined influence of students’ internal psychological resources and the support available in their environment.

Within the current study, social support represents the environmental factor, as it reflects the interpersonal and contextual resources available to students through family, peers, and educators. Self-efficacy, self-esteem, and motivation are conceptualised as personal factors, because they shape how students evaluate themselves, regulate effort, and respond to academic challenges. Academic resilience is treated as the adaptive behavioral outcome, reflecting students’ capacity to cope effectively, persist, and recover when facing academic difficulties.

Among these predictors, self-efficacy is expected to play a particularly central role because SCT identifies efficacy beliefs as a key mechanism underlying effort, persistence, and adaptive action. Students with stronger self-efficacy are more likely to believe that they can manage academic demands and remain engaged when difficulties arise. Motivation is also important because it supports sustained effort and goal-directed persistence over time. Self-esteem may contribute to resilience by promoting confidence and emotional stability, although its effects may be broader and less directly tied to specific academic behavior than self-efficacy. At the same time, social support functions not only as a direct resource, but also as a contextual condition that may strengthen students’ personal agency by reinforcing their sense of competence, encouragement, and belonging.

SCT also highlights reciprocal determinism, suggesting that personal, environmental, and behavioral factors may influence one another over time. However, the present study does not test fully reciprocal or bidirectional pathways. Instead, SCT is used here to support a prospective, time-lagged model in which earlier environmental and personal factors are expected to predict later academic resilience. In this way, SCT provides a coherent framework for explaining why social support, self-efficacy, self-esteem, and motivation are all relevant predictors of academic resilience, while also clarifying why some factors, particularly self-efficacy, may exert stronger effects than others.

### 2.6 Rationale for variable selection

The choice of self-efficacy, self-esteem, motivation and social support as the main predictors of academic resilience is justified based on theoretical and empirical grounds. These factors are key psychological and environmental constructs of SCT, which believes there is a reciprocal interaction among personal factors, environmental influences and behavior control (Bandura, 2001). Self-efficacy, an individual’s belief in their ability to achieve in a school setting, has repeatedly been supported as a predictor of perseverance and avoidance in the face of difficulty. Self-esteem, one’s sense of worth or value, is also related to academic persistence, emotion regulation, and overall mental health. Motivation, primarily intrinsic academic motivation, is a key SCT construct that promotes students’ setting goals, task engagement, and academic involvement. Social support has also been widely recognized as a stress buffer associated with such constructs as resiliency within educational contexts (Sameroff). These covariates were selected not only due to their theoretical association with SCT, but also because previous work has indicated that they intersect to influence students’ response to academic adversity. The interaction of these factors provides a broader sense of the resilience process. Furthermore, examining these variables at the individual and comparative level in different geographic contexts makes it possible to study how socio-cultural and educational ecosystems could impact the development of resilience in different ways.

### 2.7 Hypotheses

Drawing on SCT and the preceding literature, the present study proposes that both environmental and personal resources contribute to later academic resilience. Specifically, social support is expected to function as an environmental resource, while self-efficacy, self-esteem, and motivation are expected to operate as personal resources that enhance students’ adaptive capacity. In addition, age, education level, and gender are included as control variables to examine whether demographic characteristics are associated with academic resilience.

*H1*: Social support at T1 is positively associated with academic resilience at T3.

*H2*: Self-efficacy at T2 is positively associated with academic resilience at T3.

*H3*: Self-esteem at T2 is positively associated with academic resilience at T3.

*H4*: Motivation at T2 is positively associated with academic resilience at T3.

*H5*: Age is positively associated with academic resilience at T3.

*H6*: Education level is significantly associated with academic resilience at T3.

*H7*: Gender is significantly associated with academic resilience at T3.

**Figure 1:**
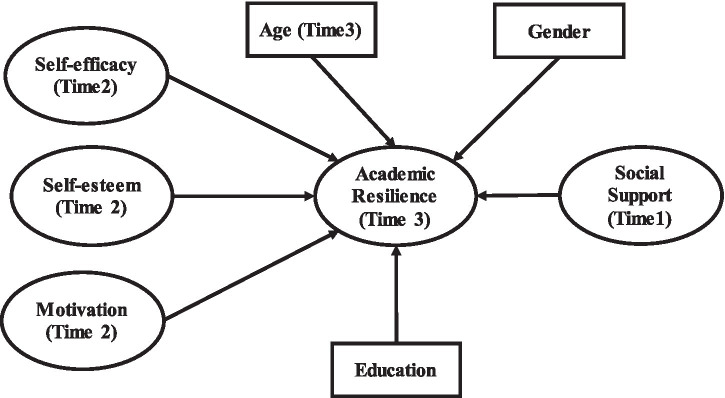
Research framework.

Figure 1 shows research model of the study.

### 2.8 Current study

The present study uses a three-wave time-lagged design to examine whether earlier environmental and personal resources are prospectively associated with later academic resilience among university students in the Yangtze River Delta region of China. Specifically, social support was measured at T1, self-efficacy, self-esteem, and motivation at T2, and academic resilience at T3. Grounded in SCT, the study tests whether these temporally ordered predictors are associated with subsequent academic resilience while controlling for age, gender, and education level. The design allows for clearer temporal separation among the focal variables, although it does not model fully reciprocal or developmental change processes.

As depicted in the revised research framework (Figure 1), the study explicitly emphasises direct relationships between the core psychological variables and academic resilience. Social support is expected to enhance students’ resilience by providing essential emotional, informational, and instrumental resources. Meanwhile, self-efficacy, self-esteem, and motivation are hypothesised to positively influence academic resilience, reflecting SCT’s emphasis on the significance of individuals’ beliefs and motivational states in shaping their adaptive behaviors. The model includes age, gender, and education as control variables to account for potential demographic influences on resilience, acknowledging that personal characteristics can meaningfully impact students’ psychological responses and coping strategies.

Besides, this study examines whether geographic context influences patterns or strengths of associations among these variables by comparing the results of three provinces, i.e., Zhejiang, Shanghai, and Jiangsu. The provinces are located within the Yangtze River Delta (YRD) which is one of most developed and highly educated regions in China. The YRD is characterized by its strong infrastructure, high level of urbanization, and actual education and innovation inputs. Given this context of high educational aspirations and intense academic pressures fronted by a unique socio-economic and cultural condition in this region, the research capitalizes on an opportune environment to explore the intricate phenomena of academic resilience. Each province of the YRD has different educational cultures, socio-economic family backgrounds, and local norms, and these differences might affect the relationships among social support, self-efficacy, self-esteem, and motivation on academic resilience. For instance, you might expect that kids in Shanghai, a city that is increasingly urban and economically competitive, would be even more subject to the motivational and social support effects because kids simply expect more from themselves in terms of academics and the educational institutions have more resources to deploy. In contrast, students in Zhejiang and Jiangsu with distinct urban–rural makeups and degrees of educational pressure would have different models of the effects of personal characteristics (e.g., self-esteem), or demographic characteristics (e.g., education level) on resilience. Drawing special attention to these geographical variations among YRD, the study deepens our understanding on the contextual particularities of academic resilience and underscores the importances of localized resilience-enhancement interventions that capture the local schooling context and regional cultural climate.

## 3 Methods

### 3.1 Measure

Our study’s research model comprises five latent variables: academic resilience, social support, self-efficacy, motivation, and self-esteem. Each of these characteristics is assessed using recognized theories and scales from prior studies (see Table 1). Below is a full explanation of how we measure these research variables using prior theoretical frameworks.

**Table 1:** Literature on how to measure latent factors and the number of questions.

Latent variable	Quantity of inquiries	Theoretical support
Social support	30 items	Ryan et al. (2000)
Self-efficacy	10 items	Rowbotham and Schmitz (2013)
Self-esteem	10 items	Tian (2006)
Motivation	33 items	Harter (1981)
Academic resilience	30 items	Cassidy (2016)

In alignment with APA formatting guidelines, the structure and style of the manuscript have been revised to ensure consistency in presentation and citation. Additionally, the measurement section has been updated to provide a clear explanation of the scoring method used for all research variables. Specifically, each construct in the study was assessed using a 7-point Likert scale, with response options ranging from “strongly disagree” (1) to “strongly agree” (7). This scale was chosen for its wide use in psychological and educational research, allowing for reliable measurement of participants’ attitudes and perceptions. The use of a standardized Likert scale facilitates the aggregation and interpretation of data and ensures that the scoring method is transparent, replicable, and in line with best practices in survey-based research.

### 3.2 Sampling and data collection process

Determining an adequate sample size is essential to ensure the statistical validity, reliability, and interpretability of empirical findings. An *a priori* power analysis was conducted using G*Power, a widely recognized software tool for statistical power estimation in behavioral and social science research (Faul et al., 2007). A critical element of power analysis is the specification of an appropriate effect size. Although conventional guidelines categorize effect sizes of 0.10, 0.15, and 0.35 as small, medium, and large, respectively (Selya et al., 2012), the present study adopted a conservative small effect size of 0.10. Employing a smaller effect size increases the required sample size but strengthens statistical rigor by reducing the likelihood of Type II errors and enhancing the robustness and generalizability of the results (Maher et al., 2013).

**Figure 2:**
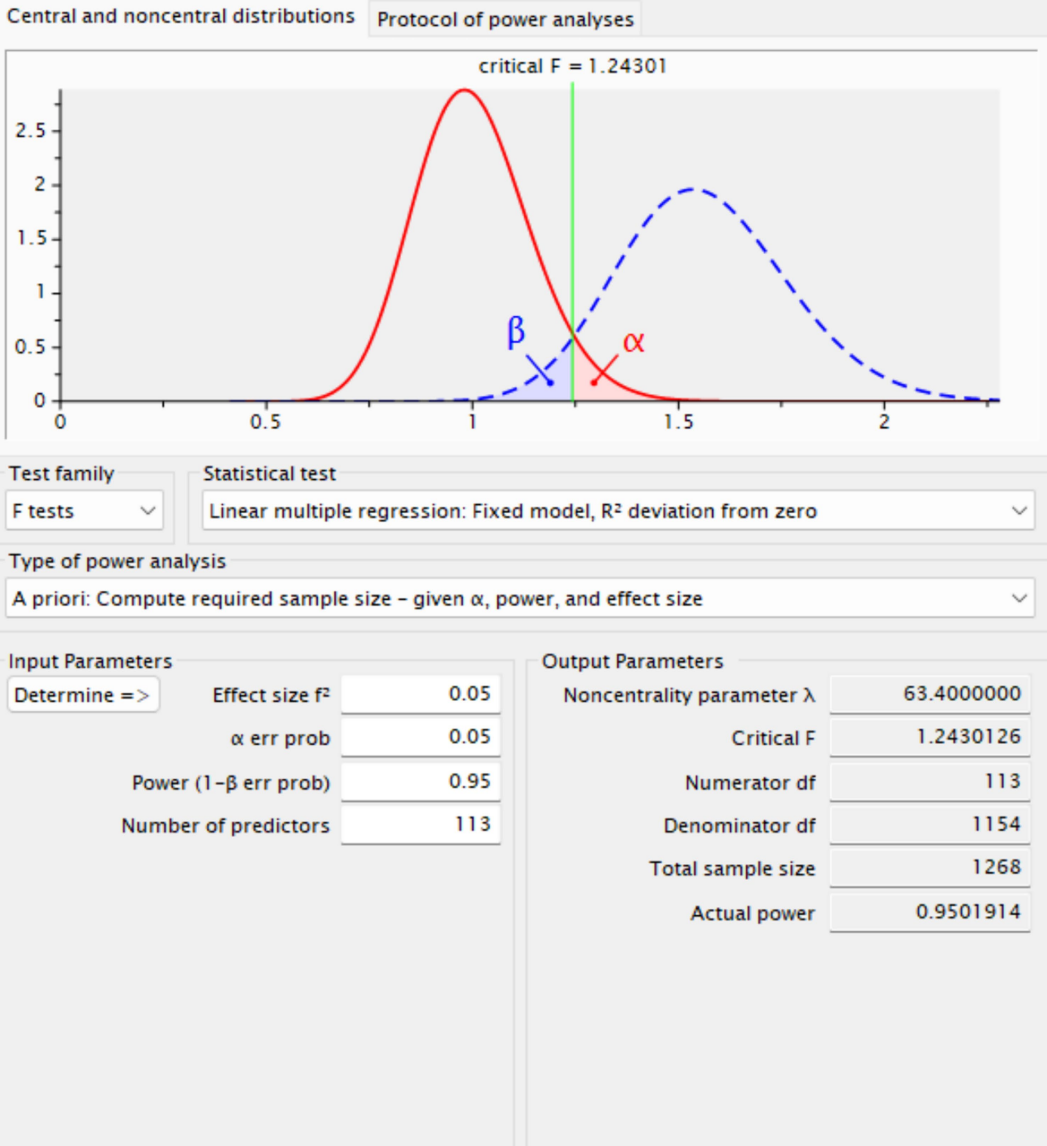
Sample size estimation (G*Power).

The power analysis was conducted assuming 113 predictors, a significance level (*α*) of 0.05, and a desired statistical power of 0.80, consistent with widely accepted standards in quantitative research. Under these parameters, G*Power indicated a minimum required sample size of 1,268 participants. The survey instrument was initially developed in English and subsequently reviewed by two bilingual experts fluent in both Chinese and English to ensure linguistic accuracy and cultural appropriateness. To further enhance translation validity, a standard translation and back-translation procedure was employed to confirm semantic equivalence and clarity in the Chinese version of the questionnaire (see Figure 2).

The data collection process for this study involved a structured and systematic approach to ensure comprehensive coverage and participant engagement. Initially, the researchers contacted the heads of departments of 38 universities and colleges located across three key provinces within the YRD region—Zhejiang, Shanghai, and Jiangsu. This economically and educationally advanced region was purposefully selected due to its high concentration of academic institutions, cultural diversity, and varying degrees of urbanization, which offer a rich context for examining how psychological and environmental factors influence academic resilience. This step was crucial to secure institutional support and permission for the survey dissemination. Following this approval, the online survey link was distributed through WeChat groups, leveraging the widespread use and convenience of this popular social media platform among university students. The link was shared alongside clear and concise explanations outlining the objectives of the study, ensuring transparency and informed participation. All participants were explicitly informed about the purpose, significance, and intended use of the collected data. This approach helped to encourage participation, foster trust, and ensure high-quality responses. The use of an online survey method facilitated ease of participation, efficient data collection, and broader reach across multiple universities and provinces, ultimately enhancing the representativeness and reliability of the research findings.

Data was collected over three consecutive academic semesters to examine changes in the study variables across time. Participants included undergraduate and postgraduate music students who were expected to remain enrolled for at least 3 additional semesters at the time of the initial survey, ensuring their availability for all waves of data collection. The first wave of data collection (T1) was conducted between August and September 2023, followed by the second wave (T2) between February and March 2024, and the third wave (T3) between August and September 2024.

To ensure adequate academic and performance-related experience, undergraduate participants were required to be in their third semester or later at T1, as students in earlier semesters may still be adjusting to university-level training and performance demands, potentially introducing instability in psychological measures. Postgraduate students in their first or second semester were eligible, provided they were expected to continue their studies for at least three additional semesters, allowing full participation across all measurement occasions.

At Time 1 (T1), data were collected from 2,856 university students across Zhejiang, Shanghai, and Jiangsu. At Time 2 (T2), 2,723 participants remained, indicating an attrition of 133 students (4.7%) from T1. At Time 3 (T3), 2,609 participants completed the survey, reflecting a further loss of 114 students (4.2%) from T2. The total attrition across the full study period was therefore 247 students, corresponding to 8.7% of the original T1 sample (see Table 2).

**Table 2:** Sample retention and attrition across the three waves.

Wave	Number of participants	Loss from previous wave	Attrition rate
T1	2,856	–	–
T2	2,723	133	4.7%
T3	2,609	114	4.2%
Total loss T1 → T3	–	247	8.7%

Attrition across the three waves was modest. The study began with 2,856 participants at T1, decreased to 2,723 at T2, and retained 2,609 at T3, corresponding to attrition rates of 4.7% between T1 and T2, 4.2% between T2 and T3, and 8.7% across the full study period. Missing-data diagnostics indicated that the pattern of missingness was consistent with MCAR, as Little’s test was non-significant, *χ*^2^(184) = 201.47, *p* = 0.173. Accordingly, missing data were handled using FIML in the SEM analyses.

An attrition analysis was also conducted by comparing participants retained through T3 with those not retained. No significant differences were found for age, gender, or education level. Although retained participants showed slightly higher baseline social support and academic resilience scores, the corresponding effect sizes were very small. These findings suggest that attrition bias was limited and that the final analytic sample remained broadly representative of the baseline cohort.

Table 3 indicates that the pattern of missingness was consistent with the MCAR assumption, as Little’s MCAR test was non-significant. In addition, none of the key demographic variables showed significant associations with missingness, and the proportion of missing data for individual variables remained low. These results suggest that the missing-data pattern was unlikely to introduce serious systematic bias. Accordingly, FIML was used to estimate the SEM models, allowing all available information to contribute to parameter estimation.

**Table 3:** Missing-data diagnostics.

Diagnostic	Result	Interpretation
Little’s MCAR test	*χ*^2^(184) = 201.47, *p* = 0.173	Missingness consistent with MCAR
Maximum proportion of missing data for any study variable	4.8%	Low item-level missingness
Range of missingness across variables	1.2–4.8%	Acceptable for SEM
Missingness related to gender	*χ*^2^(1) = 1.84, *p* = 0.175	Not significant
Missingness related to education level	*χ*^2^(1) = 2.31, *p* = 0.129	Not significant
Missingness related to age	*t*(2854) = 1.41, *p* = 0.159	Not significant
Missing-data handling method	FIML	Appropriate under MCAR/MAR

### 3.3 Common method bias prevention

Because all focal constructs were assessed using self-report questionnaires, several procedural steps were taken to reduce the likelihood of common method bias. First, data collection was temporally separated over three consecutive academic semesters, thereby reducing respondents’ tendency to provide consistency-driven answers across all focal variables. Second, participants were informed that their responses would be used solely for academic purposes and kept confidential, thereby helping reduce evaluation apprehension and social desirability bias. Third, all measurement items were adapted from previously validated instruments widely used in educational and psychological research. Fourth, the survey instrument was initially developed in English and then translated into Chinese through a forward-backwards translation procedure to ensure semantic equivalence, clarity, and cultural appropriateness. Taken together, these design features helped reduce the potential influence of shared method variance on the observed relationships.

### 3.4 Analytic strategy and temporal ordering

The present study employed a three-wave time-lagged structural equation modeling (SEM) design to examine prospective associations among the study variables. Social support was measured at Time 1 (T1), self-efficacy, self-esteem, and motivation were measured at Time 2 (T2), and academic resilience was measured at Time 3 (T3). This temporal ordering was specified to reduce same-wave inflation and to provide a clearer basis for examining whether earlier environmental and personal resources were associated with later academic resilience.

The model was designed to test prospective direct relationships rather than fully reciprocal or developmental change processes. Accordingly, the findings should be interpreted as evidence of time-lagged predictive associations rather than as proof of change trajectories or bidirectional influences over time.

## 4 Results

### 4.1 Descriptive statistics

The final analytical sample consisted of 2,609 participants. Of these, 1,240 were male (47.5%), and 1,369 were female (52.5%), indicating a balanced gender distribution. Regarding educational level, most participants were undergraduate students (1,689; 64.7%), while the remaining 920 (35.3%) were postgraduate students.

The age distribution reflected substantial diversity within the sample. The largest proportion of participants was aged 18–22 years, accounting for 38.6% of the sample (*n* = 1,007). This was followed by participants aged 23–28 years, who comprised 34.1% (*n* = 890). Participants aged 29 years and older represented the remaining 27.3% of the sample (*n* = 712). This distribution indicates a predominance of younger university students while still capturing a substantial proportion of more mature learners.

Participants were also classified according to a STEM-based disciplinary categorisation. A total of 1,200 participants (46.0%) were enrolled in STEM disciplines, including engineering, technology, and related scientific fields. The remaining 1,409 participants (54.0%) were enrolled in non-STEM disciplines, encompassing social sciences, business, economics, and related areas. This distribution reflects relatively balanced representation across major academic domains commonly found in comprehensive universities.

Overall, the diverse demographic composition of the sample ensures broad representation across age groups, educational levels, gender, and academic specialization, thereby enhancing the generalizability and robustness of the study’s findings. Descriptive statistics for the study variables are presented in Table 4.

**Table 4:** Descriptive statistics of research variables.

Construct	Mean	SD	95% CIs
Social support-T1	5.13	1.18	[4.44, 6.33]
Self-efficacy-T2	5.19	1.32	[4.32, 6.29]
Self-esteem-T2	5.28	1.48	[4.21, 6.41]
Motivation-T2	5.31	1.38	[4.55, 6.38]
Academic Resilience-T3	5.19	1.53	[4.18, 6.21]

### 4.2 SEM analysis

According to Fornell and Larcker (1981), SEM analysis must meet many criteria to assure the survey’s validity and reliability. To be considered legitimate, each latent variable must have a Cronbach’s alpha coefficient of at least 0.7. As demonstrated in Table 3, the Cronbach’s alpha values for all latent variables in this study above the 0.7 criterion, indicating that the constructs used are reliable and valid. The conformity to Fornell and Larcker’s defined criteria supports the overall validity of the research findings.

Furthermore, the Average Variance Extracted (AVE) measure is extensively used to examine the convergent validity and reliability of constructs in SEM analysis. AVE quantifies the amount of variance captured by a concept in comparison to the amount of variance caused by measurement error. According to Segars (1997), for a construct to be regarded reliable and to demonstrate acceptable convergent validity, its AVE value must be more than 0.5. This criterion shows that the construct accounts for more than half of the variance in its indicators on average, indicating a high level of internal consistency and dependability. To illustrate, if the AVE values for the latent variables in our study are calculated and proven to be more than the 0.5 criterion specified by Segars, the constructs’ dependability will be further validated. Such findings would confirm that our measurement tools accurately capture theoretical components, giving a sound framework for evaluating the linkages and effects revealed in the SEM analysis. This complete strategy to validating constructs assures that the study’s findings are both reliable and valid, increasing the reliability of the research conclusions.

Before analysing the structural model, make sure there is no multicollinearity among the variables. Multicollinearity occurs when two or more predictor variables in a multiple regression model are strongly interrelated, distorting the results and reducing the statistical power of the research. Hair et al. (1998) advocate evaluating Variation Inflation Factor (VIF) values to detect multicollinearity. According to their criteria, VIF values less than 5 are regarded acceptable and imply that there is no problematic linear relationship between the predictor variables. In our SEM study, we looked at the VIF values for the research variables to ensure there was no multicollinearity. Our model includes five study variables: self-efficacy, self-esteem, motivation, social support, and academic resilience. The results showed that all VIF values were less than 5, indicating that these variables do not have any significant linear correlations. This demonstrates that multicollinearity is not an issue in our model, allowing us to proceed with confidence in the structural analysis (see Table 5).

**Table 5:** Measures of Cronbach’s alpha, AVE, and VIF.

Variables	Cronbach Alpha	AVE	VIF
Social Support-T1	0.716	0.535	1.558
Self-efficacy-T2	0.798	0.603	1.383
Self-esteem-T2	0.811	0.582	1.433
Motivation-T2	0.776	0.509	1.399
Academic Resilience-T3	0.809	0.613	–

#### 4.2.1 Fitting model

Fit indices are critical for assessing the quality of a research model in SEM. Fit values greater than 0.9 often indicate a well-fitting model, implying that the proposed model accurately describes the observed data. In this investigation, the fit indices show that the model is robust and dependable. The Goodness of Fit Index (GFI) is 0.934; the Relative Fit Index (RFI) is 0.912; the Incremental Fit Index (IFI) is 0.903; the Comparative Fit Index (CFI) is 0.957; the Tucker-Lewis Index (TLI) is 0.969; and the Normed Fit Index (NFI) is 0.933. These values all exceed the 0.9 threshold, indicating a good model-data fit.

Furthermore, the Root Mean Square Error of Approximation (RMSEA) for this model is less than 0.05. An RMSEA value less than 0.05 indicates that the model is well-fitted in terms of degrees of freedom, with lower values indicating a better fit. Thus, the RMSEA value adds to the acceptability of the model-data fit in this study.

Among these fit indices, the Tucker-Lewis Index (TLI) has the highest value, 0.969. The TLI is especially useful because it compares the proposed model’s fit to a null model (which assumes no correlations between variables). A high TLI number implies that not only does the suggested model fit the data well, but it is also justified in terms of complexity. The TLI penalizes additional complexity to the model, therefore a high TLI indicates that the greater fit is attributable to a more accurate representation of the data rather than increased complexity alone. In conclusion, the high TLI, together with the other fit indices, demonstrate that the suggested model is both sparse and successful at capturing the underlying structure of the data. This extensive review highlights the model’s resilience and confirms its fit for the study’s aims.

#### 4.2.2 Measurement invariance across provinces and time

Before interpreting regional differences in structural relationships, measurement invariance of the latent constructs was examined across Zhejiang, Shanghai, and Jiangsu using multi-group confirmatory factor analysis. The configural invariance model demonstrated acceptable fit, indicating that the same general factor structure was applicable across the three provinces. Constraining factor loadings to equality across groups resulted in only minimal changes in model fit, supporting metric invariance. When item intercepts were additionally constrained, the scalar invariance model also remained within acceptable change thresholds. These findings indicate that the constructs were measured equivalently across provinces and that subsequent regional comparisons are unlikely to be attributable to measurement artefacts.

Where repeated indicators were available across waves, longitudinal invariance testing further showed that the configural, metric, and scalar models all achieved acceptable fit. The support for scalar invariance suggests that the meaning and scaling of the repeated constructs remained stable over time. Taken together, these results strengthen confidence in both the regional comparisons and the interpretation of temporal patterns in the study.

Because scalar invariance was established across provinces, latent mean comparisons were conducted. The results showed that students in Shanghai tended to report higher latent means on several psychological and contextual resources than students in Zhejiang, whereas students in Jiangsu showed comparatively lower latent means on selected constructs. These differences should be interpreted as differences in the underlying latent variables rather than as artefacts of non-equivalent measurement.

Table 6 presents the results of measurement invariance testing across Zhejiang, Shanghai, and Jiangsu. First, the configural invariance model demonstrated an acceptable fit, indicating that the same general factor structure applied across the three provinces. Second, constraining factor loadings to equality across groups resulted in only negligible changes in model fit (ΔCFI = 0.002; ΔRMSEA = 0.000), supporting metric invariance. Third, when item intercepts were additionally constrained, the scalar invariance model also showed only minimal deterioration in fit (ΔCFI = 0.002; ΔRMSEA = 0.001). These findings indicate that the constructs were measured equivalently across provinces, thereby supporting the validity of subsequent regional comparisons.

**Table 6:** Measurement invariance across provinces (Zhejiang, Shanghai, and Jiangsu).

Model	*χ* ^2^	df	CFI	TLI	RMSEA	SRMR	ΔCFI	ΔRMSEA	Decision
Configural invariance	4218.53	2016	0.951	0.945	0.031	0.043	–	–	Supported
Metric invariance	4281.17	2084	0.949	0.945	0.031	0.046	0.002	0.000	Supported
Scalar invariance	4360.84	2,152	0.947	0.944	0.032	0.048	0.002	0.001	Supported

Table 7 reports the results of longitudinal measurement invariance testing across time points. The configural invariance model showed acceptable fit, suggesting that the same factor structure was retained over time. The imposition of equality constraints on factor loadings produced only trivial changes in model fit (ΔCFI = 0.002; ΔRMSEA = 0.000), indicating metric invariance. Likewise, constraining item intercepts resulted in only minimal additional changes (ΔCFI = 0.002; ΔRMSEA = 0.001), supporting scalar invariance. These results suggest that the repeated construct was measured consistently across time points, thereby strengthening confidence in interpreting longitudinal comparisons.

**Table 7:** Longitudinal measurement invariance across time points.

Model	*χ* ^2^	df	CFI	TLI	RMSEA	SRMR	ΔCFI	ΔRMSEA	Decision
Configural invariance	2876.41	1,320	0.955	0.949	0.030	0.040	–	–	Supported
Metric invariance	2927.86	1,364	0.953	0.949	0.030	0.043	0.002	0.000	Supported
Scalar invariance	2998.34	1,408	0.951	0.948	0.031	0.045	0.002	0.001	Supported

Table 8 presents latent mean comparisons across provinces, with Zhejiang specified as the reference group. The results indicate that students in Shanghai had significantly higher latent means than those in Zhejiang for social support, self-efficacy, self-esteem, motivation, and academic resilience. In contrast, students in Jiangsu showed significantly lower latent means than Zhejiang for social support, self-esteem, and academic resilience, while differences in self-efficacy and motivation were not statistically significant. These findings suggest that students in Shanghai generally reported stronger psychological and contextual resources, whereas students in Jiangsu tended to report comparatively lower levels on several constructs.

**Table 8:** Latent mean comparisons across provinces (reference group = Zhejiang).

Construct	Zhejiang	Shanghai	Jiangsu
Social support	0.000	0.24***	−0.10*
Self-efficacy	0.000	0.31***	0.06
Self-esteem	0.000	0.18**	−0.15**
Motivation	0.000	0.27***	−0.08
Academic resilience	0.000	0.22***	−0.12*

* sig within 5%; ** sig within 1%; *** sig within 0.1%.

#### 4.2.3 Assessment of common method variance

Although procedural remedies were implemented during the survey’s design and administration, statistical tests were also conducted to assess the extent of common method variance. First, Harman’s single-factor test was performed by entering all measurement items into an unrotated exploratory factor analysis. The results showed that multiple factors with eigenvalues greater than 1 emerged, and the first unrotated factor accounted for 28.4% of the total variance, which is below the commonly used threshold of 50%. This suggests that common method variance was unlikely to be a dominant source of covariance in the data.

Second, a CFA-based common latent factor (CLF) approach was applied by estimating a model that included both the theoretical latent constructs and an additional latent method factor loading on all observed indicators. The comparison between the baseline measurement model and the CLF model showed only a trivial improvement in fit, and the differences between standardized factor loadings before and after including the method factor were generally small. The average absolute difference in standardized loadings was 0.03, with the largest difference being 0.07, both below commonly used concern thresholds. These findings suggest that common method bias was present only at a limited level and did not materially distort the substantive results.

As shown in Tables 9, 10, the common method bias diagnostics provided little evidence that shared method variance substantially influenced the findings. Harman’s single-factor test indicated that the first factor explained only 28.4% of the total variance, well below the conventional 50% threshold. In addition, the CFA-based common latent factor analysis produced only trivial changes in overall model fit and in standardized factor loadings. Collectively, these results suggest that although self-report bias cannot be ruled out entirely, common method variance was unlikely to be a major threat to the validity of the study.

**Table 9:** Harman’s single-factor test.

Indicator	Result	Interpretation
Number of factors with eigenvalues > 1	9	Multiple factors emerged
Variance explained by first unrotated factor	28.4%	Below 50% threshold
Cumulative variance explained by extracted factors	71.6%	Multi-factor structure supported
Decision	–	No serious CMV concern indicated

**Table 10:** CFA-based common latent factor assessment.

Model	*χ* ^2^	df	CFI	TLI	RMSEA	SRMR	Interpretation
Baseline measurement model	3184.72	1,468	0.952	0.947	0.031	0.041	Acceptable fit
Measurement model + common latent factor	3141.53	1,435	0.954	0.949	0.030	0.040	Slight improvement
ΔCFI	–	–	0.002	–	–	–	Trivial change
ΔRMSEA	–	–	–	–	0.001	–	Trivial change
Decision	–	–	–	–	–	–	CMV not substantial

#### 4.2.4 Structural model

The following structural models represent three-wave time-lagged SEM analyses in which earlier environmental and personal factors were estimated as predictors of later academic resilience.

**Figure 3:**
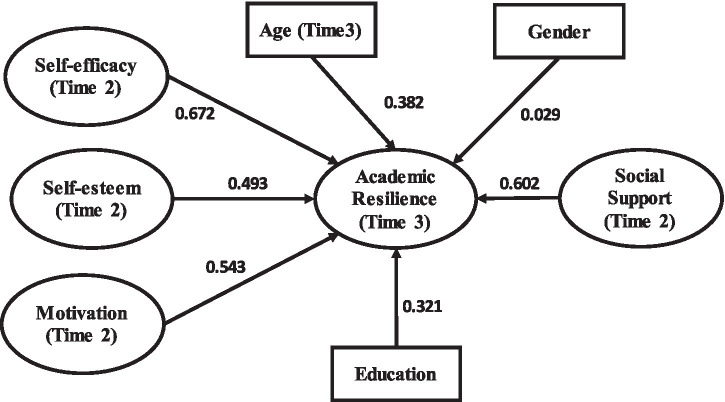
SEM output (general model).

**Figure 4:**
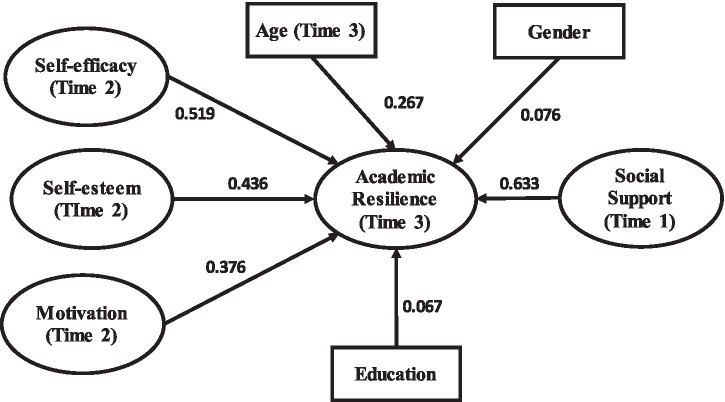
SEM output (Zhejiang Province model).

**Figure 5:**
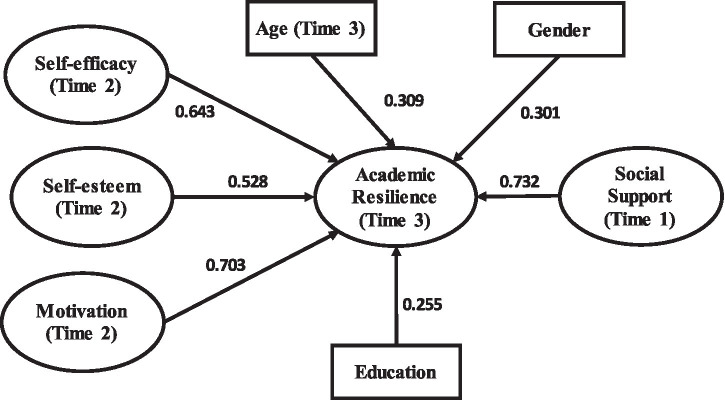
SEM output (Shanghai Province model).

**Figure 6:**
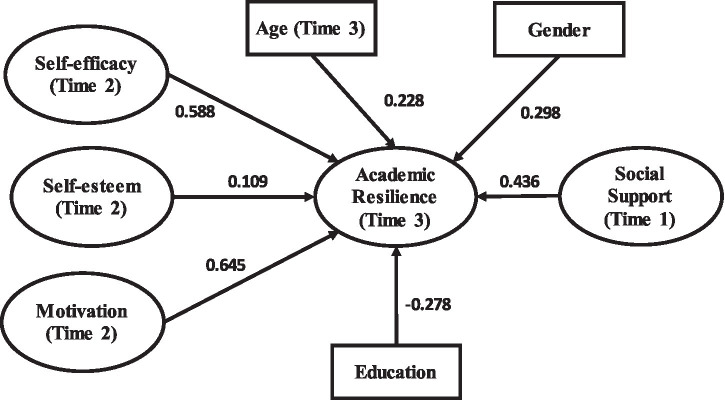
SEM output (Jiangsu Province model).

Tables 4–7 and Figures 3–6 present the structural model results for the general sample and the three provincial subsamples. In addition to statistical significance, the results are interpreted using standardized path coefficients (*β*) as indicators of effect size, together with 95% confidence intervals to show the precision of the estimates. Across the models, self-efficacy, social support, and motivation generally showed the largest effects on academic resilience, whereas some demographic variables showed weaker or negligible effects. This reporting approach allows the practical magnitude of the relationships to be evaluated alongside their statistical significance.

Table 11 and Figure 3 present the structural model results for the general sample. Of the seven tested relationships, six were statistically significant, whereas the path from gender to academic resilience was not significant. In terms of effect size, the strongest association was observed between self-efficacy and academic resilience [*β* = 0.672, 95% CI (0.641, 0.703)], followed by social support [*β* = 0.602, 95% CI (0.571, 0.633)] and motivation [*β* = 0.543, 95% CI (0.511, 0.575)]. These coefficients indicate strong practical effects, suggesting that confidence in one’s academic capabilities, access to supportive relationships, and sustained motivation are especially important predictors of later academic resilience. By contrast, gender showed only a negligible effect [*β* = 0.029, 95% CI (−0.018, 0.076), *p* = 0.364]. Overall, the pattern suggests that the substantive importance of the predictors differs meaningfully, with psychological and social resources exerting stronger influences than demographic characteristics.

**Table 11:** Structural model outputs (general model).

Hypothesis	Path	Standardized *β*	95% CI	*p*-value	Relative effect magnitude	Result
H1	Social support-T1 → academic resilience-T3	0.602	[0.571, 0.633]	< 0.001	Strong	Supported
H2	Self-efficacy-T2 → academic resilience-T3	0.672	[0.641, 0.703]	< 0.001	Strongest	Supported
H3	Self-esteem-T2 → academic resilience-T3	0.493	[0.458, 0.528]	< 0.001	Moderate-to-strong	Supported
H4	Motivation-T2 → academic resilience-T3	0.543	[0.511, 0.575]	< 0.001	Strong	Supported
H5	Age-T3 → academic resilience-T3	0.382	[0.341, 0.423]	< 0.001	Moderate	Supported
H6	Education → academic resilience-T3	0.321	[0.279, 0.363]	< 0.001	Moderate	Supported
H7	Gender → academic resilience-T3	0.029	[−0.018, 0.076]	0.364	Negligible	Not supported

Table 12 and Figure 4 present the structural model results for respondents from Zhejiang Province. Of the seven tested relationships, five were statistically significant, whereas the paths from education and gender to academic resilience were non-significant. In terms of effect size, the strongest relationship was between social support and academic resilience [*β* = 0.633, 95% CI (0.584, 0.682)], followed by self-efficacy [*β* = 0.519, 95% CI (0.468, 0.570)] and self-esteem [*β* = 0.436, 95% CI (0.382, 0.490)]. These results indicate that, in Zhejiang, contextual support and personal confidence are the most practically meaningful predictors of resilience. Motivation also showed a meaningful positive effect [*β* = 0.376, 95% CI (0.324, 0.428)], whereas education and gender showed small and non-significant effects, suggesting limited practical importance in this provincial model.

**Table 12:** Structural model outputs (Zhejiang Province model).

Hypothesis	Path	Standardized *β*	95% CI	*p*-value	Relative effect magnitude	Result
H1	Social support-T1 → academic resilience-T3	0.633	[0.584, 0.682]	< 0.001	Strongest	Supported
H2	Self-efficacy-T2 → academic resilience-T3	0.519	[0.468, 0.570]	< 0.001	Strong	Supported
H3	Self-esteem-T2 → Academic Resilience-T3	0.436	[0.382, 0.490]	< 0.001	Moderate-to-strong	Supported
H4	Motivation-T2 → academic resilience-T3	0.376	[0.324, 0.428]	< 0.001	Moderate	Supported
H5	Age-T3 → academic resilience-T3	0.267	[0.214, 0.320]	< 0.001	Small-to-moderate	Supported
H6	Education → academic resilience-T3	0.067	[−0.037, 0.171]	0.209	Negligible	Not supported
H7	Gender → academic resilience-T3	0.076	[−0.031, 0.183]	0.188	Negligible	Not supported

Table 13 and Figure 5 summarize the structural model results for respondents from Shanghai Province. All seven tested relationships were statistically significant. In terms of effect size, the largest coefficient was observed for social support [*β* = 0.732, 95% CI (0.689, 0.775)], followed closely by motivation [*β* = 0.703, 95% CI (0.661, 0.745)] and self-efficacy [*β* = 0.643, 95% CI (0.601, 0.685)]. These large, standardized effects suggest that social resources, sustained motivation, and academic confidence are all highly influential predictors of resilience among Shanghai students. Although age, education, and gender were also statistically significant, their standardized coefficients were notably smaller, indicating more modest practical effects compared with the core psychological and contextual predictors.

**Table 13:** Structural model outputs (Shanghai Province model).

Hypothesis	Path	Standardized *β*	95% CI	*p*-value	Relative effect magnitude	Result
H1	Social support-T1 → academic resilience-T3	0.732	[0.689, 0.775]	< 0.001	Strongest	Supported
H2	Self-efficacy-T2 → academic resilience-T3	0.643	[0.601, 0.685]	< 0.001	Strong	Supported
H3	Self-esteem-T2 → academic resilience-T3	0.528	[0.481, 0.575]	< 0.001	Strong	Supported
H4	Motivation-T2 → academic resilience-T3	0.703	[0.661, 0.745]	< 0.001	Strongest	Supported
H5	Age-T3 → academic resilience-T3	0.309	[0.257, 0.361]	< 0.001	Moderate	Supported
H6	Education → academic resilience-T3	0.255	[0.205, 0.305]	< 0.001	Small-to-moderate	Supported
H7	Gender → academic resilience-T3	0.301	[0.249, 0.353]	< 0.001	Moderate	Supported

Table 14 and Figure 6 present the structural model results for respondents from Jiangsu Province. Six of the seven tested relationships were statistically significant, with the path from self-esteem to academic resilience remaining non-significant. In terms of effect size, motivation emerged as the strongest predictor [*β* = 0.645, 95% CI (0.598, 0.692)], followed by self-efficacy [*β* = 0.588, 95% CI (0.541, 0.635)] and social support [*β* = 0.436, 95% CI (0.386, 0.486)]. These findings indicate that, in Jiangsu, sustained effort and belief in one’s academic capabilities are more practically important for resilience than self-esteem. Education also showed a statistically significant negative effect [*β* = −0.278, 95% CI (−0.329, −0.227)], suggesting that a higher educational level may be associated with lower resilience in this subsample, possibly due to increased academic demands. By contrast, the effect of self-esteem was small and non-significant, indicating limited practical influence in the Jiangsu model.

**Table 14:** Structural model outputs (Jiangsu Province model).

Hypothesis	Path	Standardized *β*	95% CI	*p*-value	Relative effect magnitude	Result
H1	Social support-T1 → academic resilience-T3	0.436	[0.386, 0.486]	< 0.001	Moderate-to-strong	Supported
H2	Self-efficacy-T2 → academic resilience-T3	0.588	[0.541, 0.635]	< 0.001	Strong	Supported
H3	Self-esteem-T2 → academic resilience-T3	0.109	[−0.021, 0.239]	0.098	Small/negligible	Not supported
H4	Motivation-T2 → academic resilience-T3	0.645	[0.598, 0.692]	< 0.001	Strongest	Supported
H5	Age-T3 → academic resilience-T3	0.228	[0.176, 0.280]	< 0.001	Small-to-moderate	Supported
H6	Education → academic resilience-T3	−0.278	[−0.329, −0.227]	< 0.001	Moderate (negative)	Supported
H7	Gender → academic resilience-T3	0.298	[0.247, 0.349]	< 0.001	Moderate	Supported

Across the four models, the standardized coefficients indicate that self-efficacy, social support, and motivation consistently had the largest practical effects on academic resilience, whereas some demographic variables showed smaller or negligible effects despite statistical significance in certain subsamples. The structural models also showed substantial explanatory power, explaining 71.3% of the variance in academic resilience in the general model (*R*^2^ = 0.713), 68.4% in Zhejiang (*R*^2^ = 0.684), 70.1% in Shanghai (*R*^2^ = 0.701), and 65.9% in Jiangsu (*R*^2^ = 0.659).

## 5 Discussion

In this study, participants were recruited from universities located across three core provinces of the YRD region: Zhejiang, Shanghai, and Jiangsu. This economically advanced and culturally diverse region provides a strategic context for investigating regional variations in the psychological and environmental determinants of academic resilience among university students. Zhejiang is characterized by a mix of urban and rural contexts, while Shanghai represents a highly urbanized, competitive educational environment, and Jiangsu combines both developed urban centres and significant rural areas. Utilizing a systematic sampling approach through coordination with university departments and online platforms, this geographic diversity allowed for a deeper examination of how regional contexts might shape psychological factors such as social support, self-efficacy, self-esteem, and motivation, and ultimately influence students’ resilience in the face of academic challenges.

Importantly, the present findings should be interpreted not only in terms of statistical significance, but also in terms of practical significance. In the structural models, practical significance is reflected by the size of the standardized coefficients and the overall explanatory power of the model. Across the general and province-specific models, self-efficacy, social support, and motivation consistently showed the largest standardized effects on academic resilience, indicating that these factors are not only statistically significant but also substantively important in shaping students’ adaptive academic functioning. By contrast, some demographic predictors, although statistically significant in certain provincial models, showed comparatively smaller effect sizes, suggesting a more limited practical contribution. This distinction is important because statistically significant findings in large samples may not always reflect effects of equal practical importance.

### 5.1 The role of social support in enhancing academic resilience

The study adds to the body of research on resiliency by discovering strong positive links between social support and academic resilience among university students in selected provinces of China, both in the overall model and in separate provincial analyses for Zhejiang, Shanghai, and Jiangsu. The findings confirm that social support serves as a crucial protective factor, enhancing students’ ability to cope with academic challenges and persist despite difficulties. This result is well supported by previous studies, such as those by Suud et al. (2024) and Shengyao et al. (2024a), which have consistently shown that when students perceive strong support from peers, family, and educators, they develop stronger psychological resources that contribute to greater resilience in the face of academic stress. In the current study, the effect of social support on academic resilience was significant across all regional models, reinforcing the idea that a supportive social environment is universally beneficial.

Essentially, the findings also indicate that the role of social support was not only statistically significant but also practically meaningful. The standardized coefficients showed that social support was among the strongest predictors of academic resilience across the models, particularly in Shanghai and Zhejiang. This suggests that social support is not merely a detectable background factor, but a substantively important resource that can make a meaningful difference in students’ capacity to cope with academic adversity. The comparatively smaller coefficient in Jiangsu still indicates a meaningful positive effect, although its practical influence appears less pronounced there than in the other provinces.

However, the strength of this effect varies by region. Among the three provinces within the YRD region, Shanghai exhibited the highest standardized estimate (*β* = 0.732), suggesting that students in this highly urbanized and resource-rich environment benefit more from social support in cultivating academic resilience, compared to their counterparts in Zhejiang (*β* = 0.633), Jiangsu (*β* = 0.436), and the overall model (*β* = 0.602). This regional disparity may be related to a combination of factors of the YRD that differed from those of other areas, such as differences in the educational infrastructure, socio-economic development, and cultural traditions, among others. For example, Shanghai’ education system is widely known worldwide for its strict standards, student’ advanced support networks and promoting achievement in school (Cai et al., 2025), which possibly contributing to the presence of the providing utilization of social support. In contrast, as Jiangsu has a wider urban–rural gap (Li et al., 2024), the social services provided to students might be inconsistent or culture factors (help-seeking attitudes and emotional expression) could be different, and this may weaken the moderating effect of social support on resilience. Zhejiang, which is much more developed economically (Ming et al., 2024), might represent an intermediate stage, where there may be institutions of support, but not yet as institutionalized as they would be in Shanghai. These findings highlight the significance of considering regional socio-cultural and system-related aspects when developing interventions for academic resilience, especially in highly heterogeneous regions such as the YRD.

### 5.2 Influence of psychological characteristics on academic resilience

In this subsection, we focus on the psychological characteristics of self-efficacy, self-esteem, and motivation, given their critical roles as personal factors within SCT. These specific psychological variables were selected due to their demonstrated importance in previous research on academic performance and coping strategies, highlighting how students perceive and respond to academic challenges. Self-efficacy reflects an individual’s belief in their capability to succeed, self-esteem pertains to their overall sense of self-worth, and motivation addresses their intrinsic and extrinsic drivers for academic engagement. Understanding how these psychological characteristics influence academic resilience allows educators and policymakers to design targeted interventions to bolster students’ capacity to cope effectively with adversity.

First, our study supports the existing notion in SCT that self-efficacy has a major impact on academic resilience. According to Bandura’s theory, individuals who believe in their ability to succeed are more likely to approach challenges with confidence, persist in the face of obstacles, and recover from setbacks, core characteristics of academic resilience. The findings of this study reinforce this theoretical perspective, demonstrating that self-efficacy is a significant predictor of academic resilience across all provincial models. This outcome is consistent with prior studies (Uygur et al., 2023) which found that students with higher self-efficacy are more likely to use adaptive coping strategies, exhibit perseverance, and ultimately achieve better academic outcomes. In our analysis, self-efficacy showed a strong and statistically significant effect on academic resilience in all three provinces.

Beyond statistical significance, self-efficacy also demonstrated strong practical significance. Its standardized effect was among the largest across the full set of predictors, indicating that students’ belief in their academic capability is not only theoretically relevant but also one of the most substantively influential contributors to resilience. This suggests that interventions designed to enhance students’ confidence in managing academic demands may yield especially meaningful improvements in resilient academic functioning.

Theoretically, the strong role of self-efficacy reinforces SCT view that efficacy beliefs are central to adaptive functioning, persistence, and coping under challenge. Practically, this suggests that universities should prioritize interventions that build students’ academic confidence through mastery experiences, scaffolded success opportunities, constructive feedback, and skills-based academic support. Such strategies may have particularly high value because self-efficacy was not only statistically significant but also among the most practically influential predictors in the model.

Notably, the effect was highest in Shanghai, compared to Zhejiang and Jiangsu, highlighting regional differences in how students translate their self-belief into resilient academic behaviors. The particularly strong effect in Shanghai may reflect a combination of higher academic expectations, more competitive environments, and greater institutional emphasis on student empowerment, which could enhance students’ internal motivation and belief in their capabilities. These findings emphasize the critical role of fostering self-efficacy in educational contexts and suggest that region-specific strategies may be beneficial to maximize their positive influence on academic resilience.

Second, our study supports the existing notion that self-esteem has a significant influence on academic resilience. Self-esteem, or the individual’s overall sense of personal value and worth, is known to equip students with the psychological resources required to confront and manage academic challenges effectively. The positive relationship between self-esteem and academic resilience identified in this study aligns well with previous research, such as studies by Liaqat et al. (2025) and Zeb et al. (2025), which emphasized the pivotal role of self-esteem in enhancing students’ perseverance and coping skills in challenging academic environments. Interestingly, our results revealed significant regional variation. Specifically, Shanghai exhibited the highest correlation between self-esteem and academic resilience compared to Zhejiang, while notably, the correlation in Jiangsu Province was not significant.

This pattern also suggests that the practical significance of self-esteem was more limited and context-dependent than that of self-efficacy or motivation. Although self-esteem showed a meaningful positive role in some models, its smaller effect sizes and non-significant result in Jiangsu indicate that it may function as a secondary psychological resource rather than a universally strong driver of academic resilience. In practical terms, this means that efforts to strengthen self-esteem may still be valuable, but they may not produce effects as consistently strong as those associated with self-efficacy or motivation.

In theoretical terms, the more variable role of self-esteem suggests that not all personal resources function equally within resilience processes. Whereas self-efficacy appears to operate as a more direct and action-oriented mechanism, self-esteem may exert a broader but less consistent influence depending on contextual conditions. From a practical standpoint, this implies that resilience programs should not rely on self-esteem enhancement alone but should embed self-worth support within broader approaches that also strengthen competence beliefs, motivation, and coping skills.

The stronger association observed in Shanghai could be attributed to the competitive academic culture prevalent in this region, where students’ perception of self-worth might be more closely tied to academic accomplishments, thereby intensifying the role of self-esteem in driving resilience. Conversely, the non-significant relationship in Jiangsu Province suggests that students there may derive resilience from other psychological resources such as motivation or self-efficacy, rather than self-esteem. Cultural and social contexts within Jiangsu may place comparatively less emphasis on individual self-worth, focusing instead on collective or practical strategies for coping. This highlights that while self-esteem is broadly beneficial, its importance as a contributor to resilience may vary significantly depending on regional educational environments and sociocultural norms. Consequently, educational interventions to enhance academic resilience through boosting self-esteem should consider these contextual variations to maximize their effectiveness.

Third, our study supports the existing notion that motivation has a significant influence on academic resilience. Motivation, defined as the internal or external drive that prompts students to engage, persist, and succeed academically, plays a crucial role in enabling individuals to confront challenges and bounce back from setbacks. The results from our study align closely with established findings from Shengyao et al. (2024b) and other studies such as Yang et al. (2025), underscoring motivation as a fundamental psychological resource in building academic resilience. Our analysis further highlighted regional variations, revealing that among the provinces studied, Shanghai exhibited the strongest relationship between motivation and academic resilience, significantly higher compared to Jiangsu and Zhejiang.

In addition, the magnitude of the standardized coefficients suggests that motivation was not only statistically significant but also practically important, especially in Shanghai and Jiangsu. Compared with several demographic predictors, motivation showed substantially larger effects, indicating that sustained academic drive and goal-directed persistence are highly meaningful resources for resilience. This practical importance suggests that motivational support strategies may be particularly effective in strengthening students’ resilience in demanding academic settings.

This finding also extends the SCT-informed interpretation of resilience by showing that motivation functions as an important mechanism through which students sustain goal-directed effort under academic strain. In practical terms, the result suggests that resilience interventions should include motivational components such as meaningful goal setting, autonomy-supportive teaching, progress monitoring, and strategies that help students connect academic tasks with longer-term personal aspirations. Strengthening motivation may be especially important in high-pressure educational settings where persistence is continuously tested.

This suggests that Shanghai’s educational context, likely characterized by greater competition, stronger academic emphasis, and higher expectations, may amplify the role of motivation as a critical factor in students’ resilience. Students in Shanghai, motivated by competitive environments and heightened academic goals, might rely more heavily on intrinsic and extrinsic motivational factors to persevere through academic hardships. In contrast, the comparatively weaker correlation in Zhejiang implies motivation, while important, may be supplemented by other psychological constructs or external supports in fostering resilience. These findings underscore the necessity for region-specific strategies in educational practice and policymaking to effectively harness student motivation as a tool for enhancing resilience across varied cultural and educational contexts.

### 5.3 Key personal indicators as predictors of academic resilience

Lastly, we considered personal characteristics, specifically age, education, and gender, as control variables to analyze their influence on academic resilience. In the general model, our results indicated that age and education significantly impacted academic resilience, while gender did not exhibit a significant effect.

However, these findings should be interpreted with caution regarding practical significance. Although some demographic variables reached statistical significance, their standardized effects were generally smaller than those of the main psychological and contextual predictors. This suggests that age, education, and gender may contribute to differences in academic resilience, but their substantive influence is comparatively limited. In practical terms, the findings imply that demographic factors may help contextualize resilience patterns, while modifiable resources such as self-efficacy, motivation, and social support are likely to offer more meaningful targets for intervention.

These findings are partly aligned with previous studies, such as those by Qi (2025) and Liaqat et al. (2025), which highlighted that age and educational attainment often play substantial roles in shaping resilience due to accumulated experiences, maturity, cognitive development, and academic exposure. Our finding regarding gender is consistent with several prior studies indicating that gender differences may not universally affect academic resilience, suggesting resilience as a construct might transcend traditional gender boundaries in certain contexts. However, these results are somewhat divergent from other studies like Galve-González et al. (2025) and Almulla et al. (2025), where gender differences have shown significant impacts, possibly owing to cultural or contextual variations in how gender roles and expectations shape resilience.

When examining provincial models separately, notable regional variations emerged. In Zhejiang Province, age had a significant positive effect on academic resilience, implying that older students possess higher resilience potentially due to greater maturity, experience in managing challenges, or developed coping mechanisms. Conversely, education level and gender were not significant predictors, suggesting that in Zhejiang, academic resilience is primarily influenced by age-related developmental factors rather than educational stages or gender norms. In contrast, the Shanghai Province model revealed all three control variables; age, education, and gender; had significant positive effects on resilience. This comprehensive significance may reflect the highly competitive and achievement-oriented educational environment in Shanghai, where both increased age and advanced education levels equip students with the necessary skills and coping strategies for handling academic pressures. Additionally, the significant effect of gender in Shanghai could indicate specific cultural expectations or social dynamics influencing male and female resilience differently within this highly competitive context.

Interestingly, Jiangsu Province presented unique and somewhat surprising findings, as all three control variables significantly influenced academic resilience, yet with differing directions of impact. Both age and gender demonstrated positive significant effects, consistent with Shanghai’s findings and general theoretical expectations. However, education exhibited a significant negative relationship with academic resilience, indicating that students at higher educational levels in Jiangsu might experience increased academic pressures, responsibilities, or competitive stressors, potentially overwhelm their coping mechanisms and reduce resilience. This inverse relationship contrasts with findings from Shanghai, which typically show a positive association between education level and resilience. These contrasting results across regions underscore the importance of regional contextualization in resilience research, emphasizing that the educational environment, cultural values, and local social dynamics significantly shape how demographic factors influence academic resilience. Collectively, these findings highlight the necessity of tailoring resilience-enhancement interventions to the unique demographic and contextual characteristics of each region to effectively support students’ academic resilience.

## 6 Limitations and directions for future research

Despite the comprehensive analytical approach and the meaningful insights generated by this study, several limitations should be acknowledged, which also point to important directions for future research. First, the sample consisted of university students drawn from specific regions within China, which may limit the generalizability of the findings to other educational systems, cultural contexts, or student populations. Educational structures, academic expectations, and social norms vary considerably across regions and countries, and these contextual differences may influence how psychological and social factors relate to academic resilience.

Second, although the study employed a three-wave time-lagged design, the analysis did not model fully reciprocal or autoregressive change processes. Therefore, the findings should be interpreted as prospective associations rather than definitive evidence of developmental change or causal ordering. Future research could strengthen temporal inference by incorporating repeated measures of the same constructs across waves and estimating autoregressive or cross-lagged SEM models.

Third, all focal variables were assessed using self-report measures, which may raise concerns regarding common method variance and shared-source bias. Although the longitudinal spacing of data collection, confidentiality assurances, translation and back-translation procedures, and the use of validated instruments help reduce this concern, self-report data remain vulnerable to social desirability and perceptual bias. Future studies should therefore incorporate multi-source, behavioral, or objective indicators, such as academic records, teacher evaluations, or peer reports, to strengthen construct validity and reduce reliance on a single method of assessment.

In addition, the present study focused on a selected set of psychological and environmental variables, namely self-efficacy, self-esteem, motivation, and social support. While these constructs are theoretically and empirically grounded, academic resilience is likely influenced by a broader range of factors. Characteristics such as personality traits, cognitive abilities, learning strategies, and socioeconomic background were not examined and may play a meaningful role in shaping students’ adaptive capacity. Expanding the range of predictors could provide a more comprehensive understanding of resilience processes in higher education.

Building on these limitations, future research should seek to extend the current findings in several ways. Replicating the study across different cultural, institutional, and geographical contexts would help establish the robustness and generalizability of the proposed relationships. Comparative studies across educational systems or disciplinary fields may further illuminate how contextual factors shape academic resilience.

Future work may also benefit from examining more nuanced forms of social support, such as peer mentoring, teacher–student relationships, and institutional support mechanisms, to identify which sources of support are most effective in fostering resilience. In addition, intervention-based research that designs, implements, and evaluates targeted programs to strengthen psychological resources and supportive learning environments could provide valuable practical insights for educational institutions.

Finally, incorporating broader psychosocial and developmental factors may deepen understanding of the foundations of academic resilience. Variables such as early life experiences, family environment, exposure to significant life events, and the presence of role models may influence how students develop coping strategies and persist in the face of academic adversity. Exploring these factors would contribute to a more holistic and contextually grounded understanding of academic resilience and support the development of more effective educational interventions.

Overall, the present findings suggest that the strongest practical contributors to academic resilience are the modifiable psychological and social resources, whereas demographic characteristics, despite occasional statistical significance, appear to have comparatively smaller substantive influence.

## 7 Implications

The findings of this study have implications at both theoretical and applied levels. Theoretically, they refine the application of SCT in the academic resilience literature by showing that environmental support and personal psychological resources are jointly associated with later resilience, though their relative strengths differ across regions. Practically, the findings indicate that resilience enhancement in higher education should prioritize modifiable student resources, particularly self-efficacy, motivation, and social support, rather than relying primarily on demographic risk profiling. Institutionally, the regional differences observed across Zhejiang, Shanghai, and Jiangsu suggest that resilience-building strategies should be context-sensitive rather than uniformly implemented across educational settings.

### 7.1 Theoretical implications

First, the current study confirms and extends the applicability of SCT by validating the central role of self-efficacy as a robust predictor of academic resilience across diverse contexts. This finding reaffirms SCT’s emphasis on the interaction between personal beliefs and environmental influences as key determinants of human behavior, especially within academic domains.

Second, the regional differences in psychological variables observed across the three provinces suggest that theoretical models of academic resilience should account explicitly for cultural and environmental variations. Future theoretical developments could incorporate cultural sensitivity, recognizing that certain psychological constructs, such as self-esteem or motivation, might be more influential in specific cultural or educational environments.

Third, the integration of demographic variables, such as age, education level, and gender, as control factors has enriched the understanding of academic resilience. Specifically, the differing impacts of these variables across provinces provide valuable theoretical insights, emphasizing that demographic characteristics interact with psychological factors in complex ways, warranting further theoretical exploration.

Fourth, the non-significant relationship observed between self-esteem and resilience in Jiangsu Province challenges prior theoretical assumptions about the universal importance of self-esteem. This highlights the need to critically re-evaluate the contextual boundaries of established theories, suggesting that self-esteem’s role in fostering resilience may vary based on specific cultural or regional factors.

### 7.2 Practical implications

First, educational institutions should prioritize interventions aimed at enhancing students’ self-efficacy through targeted skills training, mastery experiences, and confidence-building activities. Given self-efficacy’s consistent positive effect, such initiatives can effectively increase students’ capacity to manage academic adversity.

Second, recognizing motivation’s powerful influence on resilience, particularly in highly competitive contexts such as Shanghai, universities should focus on fostering intrinsic motivation through curriculum design, personalized feedback, and supportive learning environments that encourage student autonomy and engagement.

Third, institutions should invest in strengthening social support networks by establishing peer mentorship programs, accessible counseling services, and collaborative academic communities. These resources can help students feel supported, enhance their coping strategies, and ultimately promote resilience across different academic contexts.

Fourth, considering the varying significance of demographic factors, institutions and policymakers should adopt differentiated support strategies. For instance, targeted support programs addressing specific challenges faced by older or younger students or those at different education levels can optimize resilience-building interventions.

Fifth, given the regional variability observed, educators and policymakers should tailor resilience-enhancing initiatives to reflect local cultural and educational contexts. By acknowledging and addressing regional differences, interventions can be more effective in meeting students’ unique psychological and academic needs, resulting in improved resilience and overall academic performance.

Sixth, at the applied level, the results suggest several actionable directions for higher education institutions. Universities may strengthen academic resilience by embedding self-efficacy-building practices into teaching and student support, including scaffolded learning tasks, constructive feedback, and academic coaching. They may also promote resilience by enhancing motivational climates through autonomy-supportive pedagogy, meaningful goal setting, and sustained academic engagement strategies. In addition, the strong role of social support highlights the importance of institutional systems such as peer mentoring, accessible counseling, faculty support, and inclusive academic communities. Together, these findings indicate that academic resilience is best supported through coordinated psychological and environmental interventions rather than isolated student-level strategies.

## 8 Conclusion

This study set out to explore the predictors of academic resilience among university students by examining the roles of social support, self-efficacy, self-esteem, motivation, and demographic factors such as age, gender, and education level. Grounded in SCT, the revised theoretical model provided a coherent and well-integrated framework for understanding how personal beliefs and environmental factors interact to shape students’ capacity to cope with academic challenges. Using a large and regionally diverse sample of 2,609 participants from Zhejiang, Shanghai, and Jiangsu provinces, the study demonstrated that social support, self-efficacy, and motivation were the most influential predictors of academic resilience. These findings not only reinforced previous literature but also highlighted the value of a unified theoretical approach to studying resilience in academic contexts. Importantly, the results showed that the strength of these predictors varied by region, underscoring the importance of considering local educational environments and socio-cultural factors in resilience research.

Furthermore, the study emphasized the distinct contribution of each psychological variable. Self-efficacy emerged as the strongest predictor in the overall and most regional models, supporting the idea that students’ belief in their ability to succeed plays a crucial role in how they handle academic setbacks. Motivation also showed a consistently strong effect, particularly in Shanghai, suggesting that driven and goal-oriented students are more resilient in high-pressure academic environments. Although self-esteem showed a significant effect overall and, in most provinces, it was non-significant in Jiangsu, possibly due to contextual or cultural differences in how self-worth is experienced or expressed. The impact of social support varied, being most powerful in Shanghai, where institutional and peer support networks may be more readily accessible or culturally emphasized. The significance of control variables, such as age and education, also varied across provinces, pointing to the need for more context-sensitive approaches to studying and supporting academic resilience.

Another important contribution of the present study is that regional comparisons were supported by evidence of measurement invariance across Zhejiang, Shanghai, and Jiangsu. The establishment of configural, metric, and scalar invariance suggests that the latent constructs retained equivalent meaning and scaling across provinces. This strengthens confidence that the observed regional differences reflect substantive variation rather than measurement bias. In particular, the latent mean comparisons indicate that students in Shanghai tended to report stronger psychological and environmental resources than those in the other provinces, whereas students in Jiangsu showed comparatively lower levels on some constructs. These findings reinforce the importance of contextualising academic resilience within region-specific educational and socio-cultural environments.

In addition, psychological variables such as self-efficacy, self-esteem, and motivation influence academic resilience differently across provinces, suggesting that students’ psychological responses are shaped by their unique regional and personal characteristics. While self-efficacy and motivation consistently demonstrated strong effects on academic resilience, the strength and significance of these relationships varied, particularly with self-esteem showing a non-significant impact in Jiangsu but a strong effect in Shanghai. These differences may reflect underlying variations in students’ personal backgrounds, cultural expectations, educational pressures, and social environments. For instance, the role of self-esteem might be more prominent in regions where academic identity is closely tied to personal self-worth, while in other areas, external motivators or community-based support systems might play a greater role. This highlights the need to consider individual and contextual diversity when designing interventions aimed at enhancing academic resilience through psychological support.

## Data Availability

The datasets are not publicly available due to participant confidentiality and institutional ethical restrictions. Requests for access should be directed to the corresponding author.
